# Comparison of four polymerase chain reaction assays for the detection of *Brucella* spp. in clinical samples from dogs

**DOI:** 10.14202/vetworld.2018.201-208

**Published:** 2018-02-16

**Authors:** Eduardo J. Boeri, María M. Wanke, María J. Madariaga, María L. Teijeiro, Sebastian A. Elena, Marcos D. Trangoni

**Affiliations:** 1 Department of Diagnosis and Biological Products Production, Division of Immunology and Diagnosis, Zoonosis Institute Dr. Luis Pasteur, Av. Diaz Velez 4821 (1405), Buenos Aires, Argentina; 2 Department of Theriogenology, Faculty of Veterinary Science, Chorroarín 280 (1427), Buenos Aires, Argentina; 3 National Health Service and Food Quality (SENASA-DILAB) OIE/Brucellosis Reference Laboratory Talcahuano 1660 (1640), Martínez, Buenos Aires, Argentina; 4 Department of Biotechnology, Center for Research in Veterinary and Agronomic Sciences - National Institute of Agricultural Technology (INTA), N. Repetto y de Los Reseros, 1686, Hurlingham, Buenos Aires, Argentina

**Keywords:** *Brucella*, *Brucella canis*, canine brucellosis, clinical samples, comparison, molecular, polymerase chain reaction

## Abstract

**Aim:**

This study aimed to compare the sensitivity (S), specificity (Sp), and positive likelihood ratios (LR+) of four polymerase chain reaction (PCR) assays for the detection of *Brucella* spp. in dog’s clinical samples.

**Materials and Methods:**

A total of 595 samples of whole blood, urine, and genital fluids were evaluated between October 2014 and November 2016. To compare PCR assays, the gold standard was defined using a combination of different serological and microbiological test. Bacterial isolation from urine and blood cultures was carried out. Serological methods such as rapid slide agglutination test, indirect enzyme-linked immunosorbent assay, agar gel immunodiffusion test, and buffered plate antigen test were performed. Four genes were evaluated: (i) The gene coding for the BCSP31 protein, (ii) the ribosomal gene coding for the 16S-23S intergenic spacer region, (iii) the gene coding for porins omp2a/omp2b, and (iv) the gene coding for the insertion sequence IS*711*.

**Results:**

The results obtained were as follows: (1) For the primers that amplify the gene coding for the BCSP31 protein: S: 45.64% (confidence interval [CI] 39.81-51.46), Sp: 95.62% (CI 93.13-98.12), and LR+: 10.43 (CI 6.04-18); (2) for the primers that amplify the ribosomal gene of the 16S-23S rDNA intergenic spacer region: S: 69.80% (CI 64.42-75.18), Sp: 95.62 % (CI 93.13-98.12), and LR+: 11.52 (CI 7.31-18.13); (3) for the primers that amplify the *omp2a* and *omp2b* genes: S: 39.26% (CI 33.55-44.97), Sp: 97.31% (CI 95.30-99.32), and LR+ 14.58 (CI 7.25-29.29); and (4) for the primers that amplify the insertion sequence IS*711*: S: 22.82% (CI 17.89 - 27.75), Sp: 99.66% (CI 98.84-100), and LR+ 67.77 (CI 9.47-484.89).

**Conclusion:**

We concluded that the gene coding for the 16S-23S rDNA intergenic spacer region was the one that best detected *Brucella* spp. in canine clinical samples.

## Introduction

Brucellosis is a zoonotic infectious disease that affects animals and occasionally humans. Although canines can be affected by smooth species of *Brucella*, the most specific and epidemiologically important is the rough specie *Brucella canis* [1].

Genital discharges and abortions are the main symptoms in females; epididymitis, orchitis, and prostatitis are the main symptoms in males. Disco spondylitis and lymphadenopathy had been reported in both sexes. On the other hand, many dogs remain asymptomatic despite being infected, which makes owners unwilling to accept that their dog is ill and should not be used for breeding [2]. This disease is considered the main cause of reproductive failures in dogs, and economic losses to breeding kennels [3].

Although people are relatively resistant to infection with *B. canis*, it has been reported in laboratory accidents [4], in HIV infected people [5] and in relatives’ outbreak linked to infection in dogs [6]. People who handle dogs suspected of being infected with brucellosis should be wearing gloves to avoid the risk of exposure to *Brucella*.

Canine brucellosis still poses a dilemma for diagnosticians [7] and despite the new techniques available, it is difficult to obtain consistent results with only one test. The rapid slide agglutination test (RSAT) is a test widely used as screening [8] but must be confirmed by more specific tests such as indirect enzyme-linked immunosorbent assay (iELISA) [9] and bacteriological culture, the latter of which is considered the gold standard.

The polymerase chain reaction (PCR) has been used for the diagnosis of many infectious diseases, with different results according to the gene studied. Different gene regions of *Brucella* spp., including the gene coding for the BCSP31 protein [10], the 16S ribosomal gene [11,12], the ribosomal gene of the 16S-23S rDNA intergenic spacer region [13], the gene coding for the porins *omp2a* and *omp2b* [14,15], and the gene coding for the insertion sequence IS*711* [16,17] have been evaluated for the detection of these bacteria. In addition, many authors have used PCR assays to complement the diagnosis of brucellosis in human blood samples [18-20], canine blood samples [21], human and canine sera [22], and canine vaginal fluids and semen [23,24].

Queipo-Ortuño *et al*. [25] used a real-time PCR assay (qPCR) on 10 human clinical urine samples by amplifying the gene coding for the BCSP31 protein. More recently, we evaluated this gene for the detection of *Brucella* spp. in canine urine samples, with excellent results when compared with the bacteriological culture and the iELISA technique [26]. Kauffman *et al*. [27] evaluated canine blood, fluid, and urine samples using qPCR to amplify the *omp25* gene.

To the best of our knowledge, no comparative study of the different genes used for the detection of *Brucella* spp. in canine clinical samples has yet been done. For this reason, the aim of this study was to compare the sensitivity (S), Sp, and positive likelihood ratios (LR+) of four PCR assays for the detection of *Brucella* spp. in clinical samples from dogs. The specific objectives were to estimate the limit of detection of each PCR for each clinical sample, to estimate the index of agreement between the established gold standard and each PCR assay, to evaluate the diagnostic accuracy of each PCR assay, and to establish which assay was the most useful for the detection of *Brucella* spp.

## Materials and Methods

### Ethical approval

This research was approved by the Science and Research Committee of Zoonosis Institute Dr. Luis Pasteur, Buenos Aires, Argentina. The collection of clinical samples only required the owner´s approval as mentioned in Materials and Methods.

### Design, population, and samples

An observational comparative cross-sectional study of samples from male and female dogs of between 8 months and 9 years of age from the City of Buenos Aires and from different areas of the province of Buenos Aires, Argentina, was undertaken between October 2014 and November 2016. We did not include dogs with clinical signs of non-brucellosis-compatible diseases or those under antibiotic treatment.

Samples were taken consecutively until reaching a total of 595 samples from a balanced number of 298 sick and 297 healthy animals, working with a 95% confidence interval (CI) and a 5% error (OpenEpi program, version 3.01). The samples were processed by different operators, to ensure blind and independent measurements. Whole blood was obtained from males and females, genital fluids from females only and urine samples from males only.

After explaining the objective of the study to the animal owners, written consent was obtained for the sampling. By puncturing the external jugular vein, 6 mL of blood was taken, reserving 3 mL for blood culture, 2 mL for serology, and 1 mL for PCR. As an anticoagulant, 2.5% sodium citrate was used. Urine samples were obtained using a K-30 or K-33 catheter (depending on the size of the animal), and then 3 mL was placed in a sterile tube for bacteriology and 3 mL in an RNase-free tube for PCR, completing a total of 6 mL of urine. Vaginal fluids were collected with Dacron swabs and placed in a PCR tube containing TE buffer (10 mmol/L Tris-HCl pH 8.0, 1 mmol/L disodium EDTA pH 8.0) for storage at −4°C until processing.

### Gold standard method

The test recommended as a gold standard is “bacteria isolation,” however, due to the low sensitivity we used many combinations of different tests to be able to have positive cases.

To be able to compare the PCR assays, we followed the methodological steps for the evaluation of diagnostic tests by first defining the golden standard. To this end, we considered the ­diagnostic results found in the serological and bacteriological tests, as well as the clinical signs and epidemiology compatible with canine brucellosis. These are detailed below: (a) RSAT: The M- strain of *Brucella canis* [8] was used as a screening test, with antigens from the Malbrán Institute (Administración Nacional de Laboratorios e Institutos de Salud “Carlos G. Malbrán” - ANLIS-) or Servicio Nacional de Sanidad Animal (SENASA), Argentina. (b) RSAT with 2-mercaptoethanol (2-ME RSAT): This test was used according to the protocol previously described [8]. (c) iELISA: This assay was used according to the protocol previously described [9]. (c) Agar gel immunodiffusion test (AGID): This test was used with *B. ovis* strain REO 198, according to the protocol previously described [28]. (e) Bacterial isolation: Cultures of whole blood and urine were performed following the recommendations described [29]. (f) Buffered plate antigen (BPA) test: this test, which allows detecting anti-*Brucella* spp**. sLPS antibodies (*Brucella abortus*/S1119-3), was performed following the standard procedure [30]. (g) Compatible epidemiology: The presence of one or more of the following items was considered as epidemiology compatible with canine brucellosis: Cohabitation with positive animals; animals whose origin was a dog kennel with the previous history of brucellosis, animal shelters or streets; history of mating with untested animals; and animal boarding. (h) Clinical signs suggestive of brucellosis: Presence of orchitis/epididymitis, testicular atrophy, scrotal dermatitis, embryonic death, conception failure, abortion, perinatal death, stillborn puppies, weak pups, or spinal pain.

### Definition of healthy and sick animals

Dogs negative to serological and bacteriological tests, as well as without clinical signs or symptoms suggestive of canine brucellosis, were defined as healthy. In contrast, if dogs complied with the combination of different diagnostic tests following the ­patterns are shown in Table-1, i.e., positive bacteriology, positive AGID, symptoms plus epidemiology, RSAT plus epidemiology, RSAT plus iELISA, iELISA positive, or all of them, they were defined as sick.

**Table-1:** Combination of tests to define the established gold standard.

Gold standard	pos	pos	pos	pos	pos	pos	pos	pos	neg	neg	neg
RSAT	+	+	+ or −	+	+	−	−	+	−	−	−
2-ME RSAT	+	+	+ or −	−	−	−	−	+ or −	−	−	−
AGID	+	+	−	−	+	−	+	−	−	−	−
iELISA	+	+	−	+	−	−	−	−	−	−	−
Bacterial isolation	+	+ or −	+ or −	+ or −	+ or −	+	+ or −	+ or −	−	−	−
Clinical signs	+	+ or −	+	+ or −	+ or −	+ or −	+ or −	+ or −	−	+	−
Compatible epidemiology	+	+ or −	+	+ or −	+ or −	−	−	+	−	−	+

pos=Positive, neg=Negative, +=Positive, −=Negative, AGID=Agar gel immunodiffusion, iELISA=Indirect enzymelinked immunosorbent assay, RSAT=Rapid slide agglutination test

### Genes/gene regions amplified by PCR

Four genes were evaluated: (i) The gene coding for the BCSP31 protein (primers B4 and B5) (PCR1), (ii) the ribosomal gene coding for the 16S-23S intergenic spacer region (primers ITS66 and ITS279) (PCR2), (iii) the gene coding for porins (primers JPF and JPRca) (PCR3), and (iv) the gene coding for the insertion sequence IS*711* (primers O1 and O2) (PCR4). Table-2 [10,13,14,17,31] shows detail of sequences used to amplify the four regions.

**Table-2:** Primers used, region amplified and size of the amplicon obtained.

Primer	Sequence (5’-3’)	Target gene	Amplicon size	Author
B4	tggctcggttgccaatatcaa	BCSP31	223 bp	Baily *et al*. [10]
B5	cgcgcttgcctttcaggtctg			
ITS66	acatagatcgcaggccagtca	16s-23s rDNA intergenic spacer region	214 bp	Keid* et al.* [13]
ITS279	agataccgacgcaaacgctac			
JPF	cgcctcaggctgccgacgcaa	*omp2b omp2a*	187 bp	Imaoka *et al*. [14]
Jpr ca	cctttacgatccgagccggta			
O1	tccgcaagcttcaagccttctatcc	IS*711*	325 bp	Al Nakkas *et al*. [17]
O2	gcgtgtctgcattcaacgtaacc			
MbaF	gagaccttcaacaccccag	Exon III beta-actin gene	86 pb	Biodynamics SRL [31]
MbaR	atcacgatgccagtggtac			

### Molecular tests for detection of Brucella spp.

DNA was extracted using a High Pure PCR Template Preparation Kit Version 2.0 content version (Roche Diagnostics, GmbH Roche Applied Science, Germany), following the manufacturer’s instructions. After obtaining purified DNA, its concentration was measured with an ND1000 nanodrop spectrophotometer to determine the exact amount to be used in each PCR reaction (30-50 ng/μL). DNA was amplified in tubes with 25 μL of reaction mixture containing: 0.5 μM of each forward/reverse primer, 200 μM of each of the four deoxynucleotide triphosphates, 5 μL of 5× buffer with magnesium chloride at a final concentration of 1.5 mM per reaction (PROMEGA 5X Green GoTaq Flexi Buffer Migration Pattern) and 1 U of Taq DNA Polymerase. Primers to amplify an 86-bp segment of exon III of the beta-actin gene (mini beta-actin) were used as internal PCR control (sequence of primers 5’-3’: Forward: 5’-GAGACCTTCAACACCCCAG- 3’/Reverse: 5’-ATCACGATGCCAGTGGTA C-3’) by placing 0.1 μM of each forward/reverse primer [31].

For primers B4 and B5 [10], the thermocycling times were modified as follows: The first denaturation at 95°C for 5 min, 35 cycles at 94°C for 1 min, 62°C for 1 min, and 72°C of extension for 1 min. The final extension was at 72°C for 10 min. For primers ITS66/ITS279 and JPF/JPRca, we used the protocol previously described [13,14]. For primers O1 and O2, we modified the protocol described by Al Nakkas *et al*. [17] by increasing the number of cycles from 30 to 35. We used between 30 and 50 ng/μL of DNA according to the measurements obtained after extraction. *B. canis* strain RM6/66 at a concentration of 30 ng/μL was used as the positive control of each PCR reaction. Finally, 5 μL of ultrapure water for molecular biology was used as negative control. The amplicons obtained were resolved on 1.5% agarose gels containing 0.5 μg/mL ethidium bromide. The PCR products were analyzed by standard 1.5 agarose electrophoresis. An Applied Biosystems Veriti™ Thermal Cycler was used for the PCR reactions.

### Analytical sensitivity of each PCR assay

To determine the analytical sensitivity, 100 μL per plate of a re-suspension in Tris buffer saline (TBS) of an isolated colony of *B. canis* RM6/66 was seeded on tryptose agar and incubated at 37°C for 3 days. The bacteria were taken using 3 mL/TBS plate, collected in a 15 mL conical tube, and then separated by centrifugation at 4000 g for 5 min. The pellet was resuspended homogeneously in 4.5 mL of TBS. Finally, aliquots of 750 μL/tube were stored in 1.5 mL tubes at −80°C. The concentration of the inoculum was determined 1 week later by seeding on tryptose agar medium of 10-fold serial dilutions. After obtaining the bacterial count, which was 1.8×10^10^ colony-forming units (CFU)/mL, we determined the analytical sensitivity of the three samples collected from healthy animals, to establish the limit of detection of each PCR assay, following the recommendations described [32].

Blood samples were taken from a healthy animal and the procedure to obtain the different serial concentrations was as follows: 50 μL of the strain was placed in a tube with 450 μL of blood mixed with 2.5% sodium citrate, so a dilution of 1×10^9^ CFU/mL was obtained. Then, 50 μL of the infected blood (dilution 1×10^9^ CFU/mL) was taken and placed in a new tube containing 450 μL of blood, obtaining a dilution of 1×10^8^ CFU/mL and so on up to 1×10^−1^ CFU/mL to extract the DNA from each of the tubes with the different concentrations of *Brucella*. The protocol used for genital fluid samples was the same as that followed for blood samples, whereas for urine samples, serial dilutions were performed as in blood samples. After obtaining the different dilutions, a 1 mL aliquot of infected urine from the 1.8×10^9^ CFU/mL dilution tube was transferred to a new empty tube and centrifuged at 15,000 g for 10 min, discarding the supernatant. A new aliquot of 1 mL of urine of the same dilution was added, and this step was repeated 3 more times until the visible pellet was obtained. The pellet was resuspended in 200 μL of sterile phosphate buffered saline and then used as a sample for DNA extraction.

### Index of agreement of the four PCR assays

The index of agreement of the four PCR assays was calculated using the program Epidat 4.0, and the values of the strength of agreement were determined following the recommendations described [33].

### Statistical analysis

Statistical analysis was performed with the Epidat version 3.1 program, making 2×2 tables and receiver operating characteristics (ROC) curves. The S, Sp, and LR+ of each PCR were evaluated with the three types of samples (blood, vaginal fluids, and urine) with the respective 95% CI to compare the results [34]. To establish whether or not there were significant differences between the assays with respect to the area under the curve (AUC) of the ROC curves, analysis of variance (ANOVA) was performed using the program WinPepi version 11.43 (Copyright J Abramson).

## Results

### Samples

The number of samples was equilibrated to obtain the values of the four PCR assays tested compared with the gold standard. The best combination of test used as a gold standard in this work was RSAT plus compatible epidemiology due to the fact that the former gave 74,16% (221/298) positive samples, whereas the latter gave 84.56% (252/298). Table-3 shows different test performed on all animals. A total of 595 healthy and sick animals were evaluated, and a total of 595 PCR samples were collected: 244 blood samples, 101 urine samples, and 250 fluid samples. Samples were taken from 70.75% females (421/595) and 29.24% males (174/595). The origin of the animals was as follows: Commercial kennels 33.10% (197/595), animal shelters 9.24% (55/595), stray dogs 8.73% (52/595), and private homes 48.90% (291/595). Clinical symptoms suggestive of canine brucellosis were recorded in 9.41% (56/595) of the samples, and compatible epidemiology was found in 50.42% (300/595) of the samples.

**Table-3:** Different test performed on 595 sick and healthy dogs.

Dog’s condition	Bacteriological test		PCR test			Serological test		
		
	Blood culture	Urine culture	Blood PCR	Urine PCR	Genital fluids PCR	RSAT	iELISA	AGID

	n=595	n=121	n=244	n=101	n=250	n=595	n=50	n=64
Sick dogs	298	73	122	51	125	298	39	50
Healthy dogs	297	48	122	50	125	297	11	14

### Serological and bacteriological tests of healthy and sick animals

From the total number of animals tested, 297 were healthy, and 298 were sick (i.e., gold standard positive). All healthy animals were negative by BPA, RSAT, and blood cultures. All sick animals were next tested by BPA and RSAT. The former gave 100% (298/298) negative samples, whereas the latter detected 74.16% (221/298) of positive samples. When it was possible (80 samples due to a low quantity of serum obtained in the many samples tested.) the 2-ME RSAT was performed, showing 86.25% (69/80) of positive samples. The iELISA test, which was performed in 39 serum samples, detected 87.17% (34/39) of positive samples. The AGID test was performed in 50 serum samples, showing 62% (31/50) of positive samples. Animals were also tested by blood cultures (males and females) and urine culture (males only due to this routes of elimination are more important in males than in females) [35,36]. Results showed that 19.12% (57/298) of blood cultures were positive and that 2.01% (6/298) were contaminated, and that 16.43% (12/73) of urine cultures were positive and that 5.47% (4/73) were contaminated.

### Clinical manifestations and compatible epidemiology of sick animals

Among the 298 gold standard positive dogs, 84.56% (252/298) had compatible epidemiology, and 18.45% (55/298) had compatible clinical signs. The most prevalent symptom was abortion, followed by infertility, epididymitis, stillborn puppies, and discospondylitis with spinal pain.

### Analytical sensitivity

**Figure-1:**
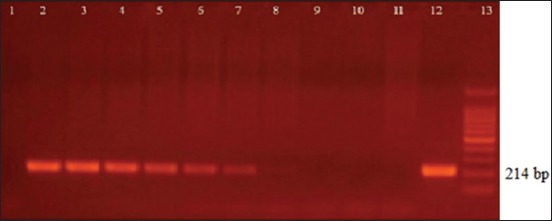
Analytical sensitivity of the ITS66/ITS279 primers blood sample. Lane 1: Negative control of DNA extraction and Lane 2-10: dilution 1.8×10^9^ to 1.8×10^1^ colony-forming units (CFU)/mL. Visible band of polymerase chain reaction (PCR) up to 1.8×10^3^ CFU/mL (lane 8). Lane 11: Negative control of PCR. Lane 12: Positive control of PCR. Lane 13: GeneRuler100 bp DNA ladder Roche xiv 11721933001.

**Figure-2:**
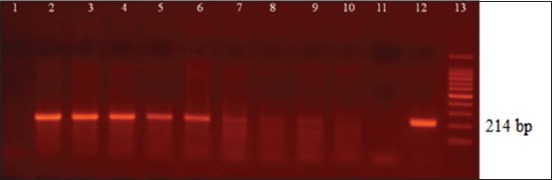
Genital fluids. Lane 1: Negative control of DNA extraction and Lane 2-10: Dilution 1.8×10^9^ to 1.8×10^1^ colony-forming units (CFU)/mL. Visible band of polymerase chain reaction (PCR) up to 1.8×10^2^ CFU/mL (lane 9). Lane 11: negative control of PCR. Lane 12: Positive control of PCR. Lane 13: GeneRuler100 bp DNA ladder Roche xiv 11721933001.

**Figure-3:**
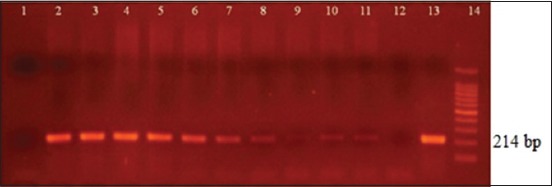
Urine samples. Lane 1: Negative control of DNA extraction, Lane 2-11: Dilution 1.8×10^8^ to 1.8×10^−1^ colony-forming units (CFU)/mL. Visible band of polymerase chain reaction (PCR) up to 1.8×10^−1^ CFU/mL (Lane 11). Lane 12: Negative control of PCR. Lane 13: positive control of PCR. Lane 14: GeneRuler100 bp DNA ladder Roche xiv 11721933001.

The limits of detection obtained, expressed in CFU/mL and discriminated by sample type, were as follows in blood, B4/B5 and O1/O2: 1.8×10^4^, ITS66/ITS279 and JPF/JPRca: 1.8×10^3^; in fluids, ITS66/ITS279: 1.8×10^2^, followed by B4/B5: 1.8×10^3^ and JPF/JPRca and O1/O2 with 1.8×10^4^; in urine samples, B4/B5: 1.8×10^−1^, ITS66/ITS279: 1.8×10^−1^, JPF/JPRca 1.8×10^−1^, and O1/O2 1.8×10^2^. The results of the primers ITS66/ITS279 are shown in Figures-1-3.

### Index of agreement of the four PCR assays

To determine the reproducibility of the PCR tests, we estimated the strength of agreement between the different PCR assays studied and the gold standard previously established. The primers B4/B5 and O1/O2 showed a moderate agreement (Kappa value 0.42 CI 0.339-0.485 and Kappa value 0.482 CI 0.431-0.535, respectively), the primer JPF/JPRca showed weak agreement (Kappa value 0.365 CI 0.291-0.440), and the primer ITS66/ITS279 showed good agreement (Kappa value 0.637 CI 0.575-0.699).

### Diagnostic S and Sp

The results of 595 samples are shown in Table-4. The S and Sp discriminated by type of clinical sample were as follows: (a) Blood: PCR 2 showed the best S, with 65.57% (CI 56.73-74.41), followed by PCR 1 with 33.61% (CI 24.81-42.40), PCR 3 with 25.41% (CI 17.27-33.54), and PCR4 with 19.67% (CI 12.21-27.14). The Sp was high in all four PCR assays, with values between 95 and 99%. (b) Fluids: PCR2 showed the best S, with 70.40% (62-78.24), followed by PCR1 with 49.60% (CI 40.44-58.78), PCR3 with 52% (CI 42.84 -61.16), and PCR4 with 28.80% (CI 20.46-37.14). Sp values were high in all four PCR assays, with values between 95 and 99%. (c) Urine: PCR2 again had the best S, with 74.83% (CI 66.16-90.70) followed by PCR1 with 64.71% (CI 50.61-78.80), PCR3 with 41.18% (CI 26.69-56.66), and PCR4 with 17.65% (CI 6.20-29.09). Sp values were high for the four assays, with values between 88 and 100%. Beta-actin was performed in all assays, however, in PCR 4 did not amplify maybe due to the annealing temperature of this PCR (69°C) compared to the other assays (60°C and 62°C).

**Table-4:** S, Sp, and LR+.

Measures of test accuracy	B4/B5 (PCR 1)	ITS66/ITS279 (PCR 2)	JPF/JPR ca (PCR 3)	O1/O2 (PCR 4)
S	45.64% (CI 39.81-51.46)	69.80% (CI 64.42-75.18)	39.26% (CI 33.55-44.97)	22.82% (CI 17.89-27.75)
Sp	95.62% (CI 93.13-98.12)	93.94% (CI 97.06-96.82)	97.31% (CI 95.30-99.32)	99.66% (CI 98.84-100)
LR+	10.43 (CI 6.04-18)	11.52 (CI 7.31-18.13)	14.58 (CI 7.25-29.29)	67.77 (CI 9.47-484.89)

PCR=Polymerase chain reaction, CI=Confidence interval, S=Sensitivity, Sp=Specificity, LR+=Likelihood ratios

## Statistical analysis of the results

**Figure-4:**
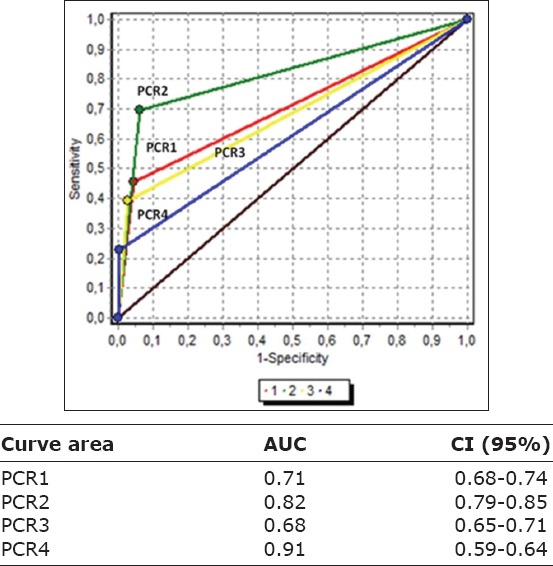
Receiver operating characteristics curves of the four polymerase chain reaction assays with the area under the curve and their respective confidence interval of the 595 samples evaluated.

To analyze the increase in the chance that the dog is ill if the PCR was positive, we determined the LR+. The values found for LR+ did not yield consistent results. On the other hand, because of a difference found in the CI of the AUC of PCR2, an ANOVA test was performed to determine whether or not there were significant differences between assays. p-value was <0.01 among all PCR combinations, except when comparing PCR1 versus PCR3 (p>0.05), between which there were no significant differences. Figure-4 shows the four ROC curves of PCR assays.

## Discussion

This kind of study needs measuring positive and negative results and so, we took samples from kennels with and without brucellosis, shelters (because these places have risks of brucellosis because there is no control of each animal) and finally, dogs with owners who had dogs adopted from the street.

The microbiological isolates obtained in this study showed that, although bacteriological culture is specific for diagnosis of canine brucellosis, it has low sensitivity. Besides, there are risks for the operator. The RSAT showed S levels similar to those described in other studies [13,27], whereas the iELISA was positive in some cases where the reaction to the other tests was negative. According to Lucero *et al*. [9], this result is not considered a false positive. This may have been due to the fact that iELISA detects antibodies earlier than the RSAT [2].

In this work, four genes were compared for detection of *Brucella* spp.: The gene coding for the BCSP31 protein, 16s-23s rRNA (ribosomal interspacer), IS*711* (insertion sequence), and *omp2a* and *omp2b* (outer membrane genes), which were contrasted with a pre-established gold standard method. The method of DNA extraction was that of commercial extraction columns, which, theoretically, reduces the problems of inhibition of the reaction.

The cases in which the PCR was positive without detection by the other combinations of gold standard could be explained by a possible contact of the dogs with *Brucella* spp. at doses lower than those required for the development of the disease (minimum conjunctival infectious dose 1×10^4^ and 1×10^6^ CFU/mL orally) [1]. In this case, no detectable antibodies would have been generated with the serological tests and the dogs would not have shown compatible clinical symptoms.

The gene coding for BCSP31 has been studied by many authors, with excellent S and Sp results. The primers used in this work were those previously designed [10], which were originally tested on *Brucella* cultures. Subsequently, many researchers evaluated them by using human blood samples, with very good results [19,20]. In this work, the results obtained showed a moderate agreement with respect to the gold standard and the values of S and Sp were different from those obtained by those authors.

The gene coding for BCSP31 has also been evaluated for the detection of *Brucella* spp. in human urine samples [25]. Recently, we have also evaluated it in male canine urine, with excellent results when tested with urine cultures and iELISA [26].

Unlike that found in the present study when using the ribosomal gene of the 16S-23S rDNA intergenic spacer region, the Brazilian researchers [13] found very high values of S and Sp in blood samples. This may be explained by the fact that the DNA extraction method used in our study was that of extraction columns versus phenol chloroform in the work of Keid *et al*. [13]. The antigen used by Keid *et al*. for the serological test of RSAT was different from the one used in this study. In our work, we used the *B. canis* strain M-, which reduces the number of false positive results by 50% [8], whereas the *B. ovis* strain [37] used by the Brazilian researchers is not the one recommended as a screening test for canine brucellosis. All this would lead to substantial differences in the results obtained.

In samples of genital fluids from female dogs, Keid *et al*. [23] obtained the same analytical sensitivity results as those in blood samples. The difference of the results of the work of the Brazilian researchers is that the gold standard method was the same PCR combined with blood and/or fluid results. Although not consistent with previous studies, the primers designed by the Brazilian researchers yielded very well S/Sp values with their respective CIs.

Leal-Klevezas *et al*. [15] developed a diagnostic PCR amplifying the *omp2b* region of *B. abortus* and obtained very good results using the JPF/JPR ­primers. In the present study, the primers JPF forward and JPRca reverse, developed by Imaoka *et al*. [14], were used (PCR3). The values of S and Sp were in third place after those of PCR 2 and PCR1, thus showing a low usefulness for clinical samples. However, very good results have been obtained with the JPF/JPRca primers in *Brucella* culture [14,38].

Al Nakkas *et al*. [17] used specific primers for the amplification of IS*711* with S and Sp values of 100% in human blood samples. Nevertheless, these primers showed low S for detection of clinical samples from dogs. One of the possible causes of the results obtained when using the IS*711* insertion sequence could be due to the low number of copies present in *B. canis* compared to other *Brucella* species such as *B. melitensis*, *B. abortus*, or *B. ovis*.

The LR+ shows the increase in the chance that the individual is ill if the PCR is positive and has the advantage of not being influenced by the prevalence of the disease. Values greater than or equal to 10 show a strong probability of having the disease if the test (PCR) is positive [34]. Table-4 shows that all the tests gave LR+ results between 10 and 14. Furthermore, the CIs overlapped in the four assays, independently of the sample analyzed, with values very far from each other. The LR+ is a ratio and, by showing the overlap between the four assays, this indicator alone was not used to show the usefulness of the PCR tests. For this reason, we used the AUC analysis of the ROC curves.

Diagnostic accuracy can be measured by analyzing the AUC of the ROC curves, which are shown in Figure-4**.** The ROC curves give a visual idea of the overall performance of a test. As we can see, the four ABCs allowed evaluating which of the PCR assays was the best in the present study, after the use of the statistical ANOVA test. By means of the ROC curves, a better performance of one of the tests could be determined graphically.

## Conclusions and recommendations

PCR2 showed the best sensitivity in the detection of *Brucella* spp. in canine clinical samples followed by PCR1, PCR3, and PCR4, respectively. Sp was high in all four assays, and LR+ did not yield conclusive results. The analytical sensitivity of PCR2 in clinical samples was the one that had the highest percentage of detection in canine clinical samples of blood, genital fluids, and urine. PCR2 showed a good concordance with the old standard method with respect to the other three assays. Therefore, PCR2 is as useful as the established gold standard and is recommended to use it together with the gold standard. The area under the ROC curve of PCR2 was higher than that of the three remaining assays. Thus, PCR2 had better diagnostic accuracy than the other PCRs tested. The analysis of the CIs using ROC curves added to the significant differences of PCR2 respect to the other PCR tests (p<0.01), determining its greater usefulness in the detection of *Brucella* spp. in the canine clinical samples evaluated.

According to our results, we recommend for canine brucellosis diagnosis the following strategy: RSAT and epidemiology as a gold standard, with or without bacterial isolation. The mainly clinical samples for PCR in male dogs are blood and urine and in female dogs: Blood and vaginal fluids. In all suspected cases of brucellosis, many tests such as RSAT, PCR, and the microbiological test should be performed.

## Authors’ Contributions

EJB and MDT designed all steps of the study and wrote the manuscript. EJB, MMW, MJM, and MLT collected samples. MJM, EJB, and MLT made all PCR assays. SAE contributed to the microbiological and serological test. EJB, MDT, and MMW revised the results and discussed all items. All authors read and approved the final manuscript.
